# Activated microglia contribute to neuronal apoptosis in Toxoplasmic encephalitis

**DOI:** 10.1186/1756-3305-7-372

**Published:** 2014-08-15

**Authors:** Yi-hua Zhang, He Chen, Ying Chen, Lu Wang, Yi-hong Cai, Min Li, Hui-qin Wen, Jian Du, Ran An, Qing-li Luo, Xue-long Wang, Zhao-Rong Lun, Yuan-hong Xu, Ji-long Shen

**Affiliations:** The Key Laboratory of Zoonoses and Pathogen Biology Anhui, and Department of Parasitology, Anhui Medical University, Hefei, China; Clinical Laboratory, the First Affiliated Hospital of Anhui Medical University, Hefei, Anhui China; Department of Blood Transfusion, the First Affiliated Hospital of Anhui Medical University, Hefei, Anhui China; Department of Biochemistry and Molecular Biology, School of Basic Medicine, Anhui Medical University, Hefei, China; State Key Laboratory of Biocontrol and Center for Parasitic Organisms, School of Life Sciences, Zhongshan School of Medicine, Sun Yat-Sen University, Guangzhou, China

**Keywords:** Toxoplasmic encephalitis, Microglia, Neuronal apoptosis, Minocycline

## Abstract

**Background:**

A plethora of evidence shows that activated microglia play a critical role in the pathogenesis of the central nervous system (CNS). Toxoplasmic encephalitis (TE) frequently occurs in HIV/AIDS patients. However, knowledge remains limited on the contributions of activated microglia to the pathogenesis of TE.

**Methods:**

A murine model of reactivated encephalitis was generated in a latent infection with *Toxoplasma gondii* induced by cyclophosphamide. The neuronal apoptosis in the CNS and the profile of pro-inflammatory cytokines were assayed in both *in vitro* and *in vivo* experiments.

**Results:**

Microglial cells were found to be activated in the cortex and hippocampus in the brain tissues of mice. The *in vivo* expression of interleukin-6 (IL-6), interleukin-1β (IL-1β), tumor necrosis factor-α (TNF-α), and inducible nitric oxide synthase (iNOS) were up-regulated in TE mice, and accordingly, the neuronal apoptosis was significantly increased. The results were positively correlated with those of the *in vitro* experiments. Additionally,apoptosis of the mouse neuroblastoma type Neuro2a (N2a) remarkably increased when the N2a was co-cultured in transwell with microglial cells and *Toxoplasma* tachyzoites. Both *in vivo* and in *vitro* experiments showed that minocycline (a microglia inhibitor) treatment notably reduced microglial activation and neuronal apoptosis.

**Conclusions:**

Activated microglia contribute to neuronal apoptosis in TE and inhibition of microglia activation might represent a novel therapeutic strategy of TE.

## Background

*Toxoplasma gondii* is an obligate intracellular parasite which infects nearly 30% of the human population worldwide [1, 2]. Infection of immunocompetent individuals with *T. gondii* does not result in dominant clinical disease. In immunocompromised individuals, however, such as HIV/AIDS or those with continuous immunosuppressive medication, may have clinical manifestations or even life-threatening Toxoplasmic encephalitis (TE) due to reactivation of the chronic quiescent infection [3, 4]. For those patients, dormant encysted bradyzoites change into fast-replicating tachyzoites and cause severe disease and even death by damage to the brain [5]. TE is the most vital outcome of toxoplasmosis in immunocompromised patients who may have fever, headache, altered mental state, seizures, weakness, cranial nerve disturbances, sensory abnormalities, cerebellar signs, meningismus, movement disorders, and neuropsychiatric disorders as well [2, 6]. Previous studies have demonstrated that inflammation potentially is one of the principal contributors to neuronal damage and death in chronic *T. gondii* infections [4, 7, 8]. So far the underlying mechanism of microglia-involved pathogenesis in TE has not been well elucidated. Microglia are the resident immune cells in the CNS that would be activated in response to injury, inflammation or the presence of pathogens [9, 10]. Microglia can secrete anti-inflammatory cytokines and neurotrophic factors that are potentially neuroprotective [11]. Activated microglia, however, could significantly induce production of a large array of inflammatory cytokines, like interleukin-1β (IL-1β), tumor necrosis factor alpha (TNF-α) [12] and inducible nitric oxide synthase (iNOS) [13], leading to the neuronal cell damage of inflammatory surroundings. The bystander effect of activated macroglia plays a decisive role in the pathogenesis of traumatic brain injury (TBI), intracerebral hemorrhage (ICH), manganese poisoning, and Lyme neuroborreliosis (LNB) [14–17].

Without the involvement of lipopolysaccharide (LPS), *T. gondii* glycosylphosp- hatidylinositols (GPIs), which are known to activate TLR2 and TLR4 receptors, may trigger production of inflammatory cytokines by mouse macrophages [18]. Additionally, *Toxoplasma* secretes effector molecules of rhoptries(ROPs) and dense granules(GRAs) that have been shown to stimulate inflammatory mediators in macrophage [12, 19].

Accumulated evidence shows that *T. gondii* may not directly cause the apoptosis of host cells [20, 21], but indirectly renders the cells vulnerable to immune-mediated responses [21]. Infection of *T.gondii* results in increased levels of inflammatory mediators [22] and microglial nodules [23] in the CNS. Multitudinous studies have focused on the characteristics of systemic immunity in control of TE, and little is known about the role of microglia in the immunopathogenesis of reactivated TE. Herein we unfolded the functions of microglia and revealed the effect of activated microglia in neuronal apoptosis of TE.

## Methods

### Parasites and cell lines

*T.gondii* Wh6 strain with genotype Chinese 1 (ToxoDB^#^9) was isolated as previously described [24]. Cysts were maintained in the brain of chronically infected mice for *in vivo* infection, whereas tachyzoites were grown in human foreskin fibroblast (HFF) monolayers for *in vitro* experiments. The BV-2 mouse microglial cell line and mouse neuroblastoma Neuro2a (N2a) were kindly provided by Dr. SH Huang (Department of Immunology, Anhui Medical University) and cultured in Dulbecco’s modified eagle’s medium (DMEM) supplemented with 10% fetal bovine serum (FBS, GIBCO), 100U/ml penicillin, 100 μg/ml streptomycin, and 2 mM L-glutamine.

BV-2 cells were seeded at 1 × 10^6^ cells/well in a 12-well plate. After 2 h, the medium was replaced by 1% FBS and co-cultured with fresh *T. gondii* tachyzoites at an MOI of 1:1 for 24 h.

### Animals and infection

Animal use in all experimental procedures was approved by the Institutional Animal Care and Use Committee of Anhui Medical University. BALB/c mice (4–6 weeks old) were randomly assigned into three groups: the control group (Control), the group of Toxoplasmic encephalitis (TE), and the group of TE with minocycline treatment(TE + M). The mouse model of TE was established as previously described with slight modifications [25, 26]. Briefly, the cysts from Wh6 strain were prepared by homogenization of the brain tissues in phosphate-buffered saline (PBS). BALB/c mice were intragastrically administered with 30 cysts. After 45 days, mice with latent infection were intraperitoneally given with cyclophosphamide (50 mg/kg, Baxter Oncology GmbH, Germany) to induce recurrence of toxoplasmosis. Simultaneously, mice were administered with minocycline (45 mg/kg, Sigma) and cyclophosphamide (50 mg/kg) intraperitoneally 45 days post-infection for inhibition of microglia activation. Fifteen days later, all mice of the three groups were euthanized for collection of the brain parenchyma and for subsequent experiments.

### Enzyme-linked immunosorbent assay (ELISA)

IL-1β, IL-6, and TNF-α were measured with a commercial kit according to the manufacturer’s instructions (4A Biotech Co, Beijing). The supernatant was collected from brain tissue homogenization and BV-2 was co-cultured with *T.gondii* tachyzoites. Brain tissues (100 mg) from each mouse were homogenized and centrifuged at 12 000 g for 15 min. Supernatant was harvested from BV-2 cells co-cultured with tachyzoites (MOI 1:1) for 24 h.

### Immunoblotting

Proteins were extracted from brain tissues (100 mg), BV-2 cells (1 × 10^5^), and N2a cells (1 × 10^5^), respectively, and were subjected to SDS-PAGE. Separated proteins were transferred onto nitrocellulose membranes (Millipore Corp, Billerica, MA) and were blocked with 5% skimmed milk. After blocking, the membranes were incubated with the primary antibodies, including mouse monoclonal anti-NeuN (1:1 000, Chemicon, Temecula, CA), mouse monoclonal anti-iNOS (1:500, BD, Bio-science), and mouse monoclonal anti-actin (1:500, Santa Cruz, CA). After washing, the membranes were incubated with horseradish peroxidase (HRP)-conjugated secondary antibodies (1:10 000, Zhongshan BioTech Co, China). The protein bands were visualized by using an ECL kit (Super Signal West Pico, Thermo Scientific, USA).

### qRT-PCR for cytokines detection

Total RNAs from brain tissues and BV-2 cells were extracted by using TRIzol reagent (Invitrogen, USA, CA) and reverse-transcribed with cDNA reverse transcription kit (Invitrogen, USA, CA). Semiquantitative PCR was run for cytokine (TNF-α, IL-6, and IL-1β) detection. Primer design and PCR amplification were performed as previously described [27].

### Parasite burden

The mice were euthanised and the brain tissues were harvested. To quantify the parasite burden in the brain of each mouse, a 50 mg brain tissue sample taken from each mouse in the respective groups was digested and DNA templates were extracted by using a DNA extraction kit (QIAGEN GmbH, Hilden, Germany) following the instructions of the manufacturer. The concentration or yield of DNA was determined with UV spectrophotometry based on an OD ratio at 260 and 280 nm (an absorbance ratio of 1.8-2.0). Subsequently, an ABI prism 7500 sequence detection system (Applied Biosystems, Foster City, CA, USA) was used for 40 cycles of polymerase chain reaction (PCR). The cycle threshold values (Ct) indicative of the quantity of the target gene expressed were determined. In this assay, an increase of fluorescent signal above a preset threshold within 38 PCR cycles was considered positive (i.e., Ct < 38). The thermal cycling conditions were programmed according to the manufacturer’s guidance.

### Histopathology

Brain tissues were harvested from mice with chronic *T.gondii* infection and TE and fixed in 10% formalin, embedded in paraffin and cut into 4 μm-thick sections. The sections were stained with hematoxylin and eosin. Histopathology and immunohistochemical studies were carried out to assess TE models based on the diagnostic criteria of TE released by the US Centers for Disease Control and Prevention (CDC) [28].

### Immunohistochemical staining

Immunohistochemical staining was performed as previously described [29] for detection of parasites and microglia. Brain sections were stained with mouse monoclonal anti-*Toxoplasma* SAG1(1:100, Abcam, Cambridge, UK) and rabbit polyclonal anti-Iba-1(1:1000, Wako, Osaka, Japan) at 4°C for overnight. After rinsing with PBS for 3 × 5 min, the sections were incubated with goat anti-mouse IgG and goat anti-rabbit (Zhongshan BioTech Co, China) at 1:75 dilution for 1 h at room temperature, and then with peroxidase-antiperoxidase-coupled secondary antibodies for 30 min. Finally, the sections were developed with a mixture of 3,3’-diaminobenzine(DAB) and counterstained with hematoxylin. The iNOS was detected using multiple fluorescence immunohistochemistry *in vivo* and *in vitro*. Frozen sections of mouse brain tissues were prepared as previously described [30]. The sections were co-stained with the primary antibodies, including mouse monoclonal anti-iNOS (1:500, BD Bioscience, NJ), rabbit polyclonal anti-Iba-1(1:1000, Wako, Osaka, Japan), FITC-mouse monoclonal anti-*Toxoplasma* SAG1(1:100, ViroStat, Portland, ME, USA), rabbit polyclonal anti-caspase-3(1:500, Santa Cruz, CA), at 4°C for overnight. After rinsing with PBS for 3-5 min, the matching secondary antibodies (anti-mouse -FITC or anti-rabbit-Rhodamine, 1:100, Zhongshan BioTech Co, China) were added to the slides and left for 1 h at room temperature. After rinsing with PBS, the sections were incubated with Hoechst for 10 min, and then were observed under fluorescence microscope. To quantify the number of microglia cells labeled with Iba-1, six sections per mouse (from cortex and hippocampus) were examined with microscopy. The images were analyzed with Image Pro Plus 6.0 software.

### TUNEL assay

For TUNEL assay, N2a cells were used for an *in vitro* experiment. Transwell culture was used to physically separate mouse N2a cells from BV-2 cells. The N2a cells were plated directly into 12-well tissue culture plates at a density of 5 × 10^5^ parasites and BV-2 cells were plated on transwell filters (0.4 μm pore size; Corning) and then incubated for 24 h at 37°C and 5% CO_2_ in DMEM with 1% FBS. To assess neuroprotective effects, Wh6 tachyzoites and BV-2 cells were plated on transwell filters and treated with minocycline (Sigma, 45 μM). The cells were fixed with 4% paraformaldehyde for 30 min and permeabilized with PBS containing 0.4% Triton X-100 for 15 min. After blocking with 4% BSA, the cells were then incubated in TUNEL kit (Roche Diagnostics GmbH, Mannheim, Germany) according to the manufacturer’s instructions. To assess apoptosis, the numbers of TUNEL stained nuclei were counted by two investigators who worked blindly with regard to the treatments in five randomly selected microscopic fields at × 400 magnification per section.

### Statistical analysis

SPSS (the Statistical Package for Social Sciences) 13.0 (SPSS Inc, Chicago, IL) was used for data analysis. All data are expressed as mean ± SEM. Differences between groups were assessed by one-way ANOVA and the SNK multiple comparison post-test or Student’s *t* test. Differences were considered statistically significant when p < 0.05.

## Results

### *Toxoplasma*encephalitis model was established

**Figure 1:**
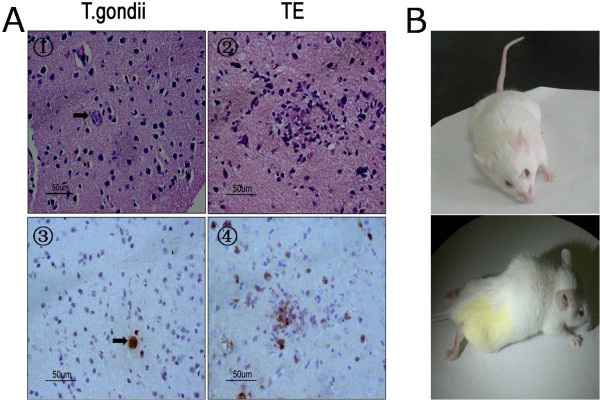
***Toxoplasma*** **encephalitis model was established.** Microscopic observation of the brain tissues of mice latently infected with cyst-forming *T. gondii* Wh6 strain (genotype Chinese 1) reactivated by cyclophosphamide administration. **(A①)** and **(A③)**, a single cyst (arrows) in the cortex of chronically infected mice; **(A②)** necrosis and inflammatory infiltration noted in foci of recrudescence; **(A④)** free tachyzoites in TE (×400). The sections were incubated with mouse anti-*Toxoplasma* SAG1 monoclonal antibody and peroxidase-antiperoxidase-coupled secondary antibody, followed by DAB colorization. Figure **B**, hemiplegia and paraplegia in model mice with *Toxoplasma* encephalitis.

Mice with latent *Toxoplasma* infection were immunologically compromised by cyclophosphamide administration for 15 days and, accordingly, TE pathology in the brain tissues of animals was observed. Histological and immunohistochemical analyses showed that necrosis (Figure 1A②) and free tachyzoites (Figure 1A④) were present in the cortex of immunosuppressed mice. Inflammatory infiltration of neutrophils and lymphocytes was seen in foci of free tachyzoites. Inversely, no infiltration of inflammatory cells in the surrounding tissues of the single cyst was noted in the brain sections of mice with chronic *Toxoplasma* infection (Figure 1A① and A③). Subsequently, we compared the parasite loads in the brain tissues of TE and TE + M to exclude the parasiticide effect of minocycline. As a result, no statistical significance of *Toxoplasma* DNA quantification was seen between TE and TE + M groups (data not shown). Hemiplegia, paraplegia, and other CNS injuries developed in mice with TE (Figure 1B).

### *T. gondii*infection induced neuronal apoptosis in TE that was inhibited by minocycline treatment

**Figure 2:**
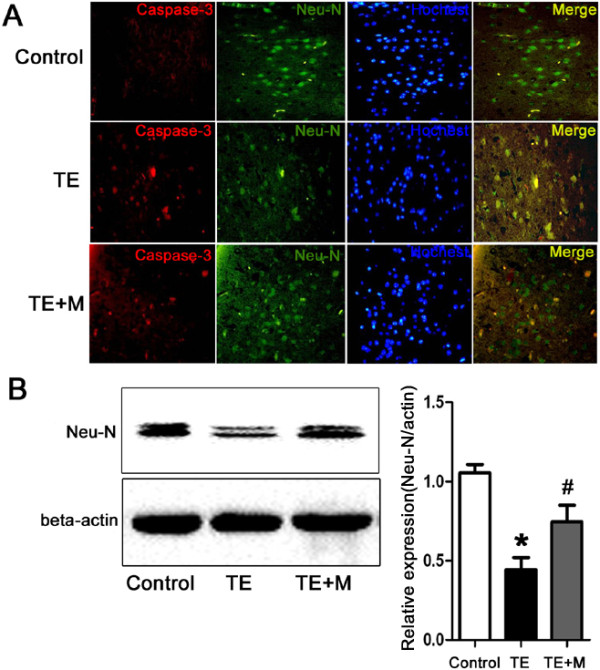
**Neuronal apoptosis in the CNS of control, TE, and TE + M mice. (A)** Double immunofluorescent staining of caspase-3 (a marker of cell death) and NeuN (a specific marker of neuron cell) demonstrated apoptosis of neuron cells. Brain sections from cortex of control, TE and TE + M animal groups were stained with polyclonal caspase-3(red) antibody and monoclonal NeuN antibody (green). Cells were counter-stained with Hoechst to show nuclei (blue). Double immunostaining showed caspase-3 expression in neurons. Increased number of caspase-3 positive cells in TE was reduced significantly in TE + M. **(B)** Western blotting assay was used for analysis of protein NeuN expression in control and TE and TE + M mice. It showed that the downregulated expression of NeuN in TE was increased significantly after treatment with minocyline (TE + M). Values represent the means ± SEM from six animals in each group (*, p < 0.01 *vs*. Control. #, p < 0.01 *vs*. TE).

**Figure 3:**
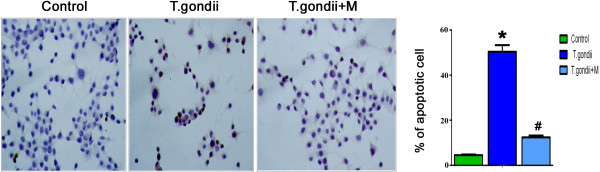
**TUNEL assay for analysis of apoptosis of mouse neuronal cells (N2a).** A small number of cell death was observed when N2a was co-cultured with BV-2. The number of TUNEL positive cells increased when N2a was co-cultured with *T.gondii* tachyzoite-infected BV-2 cells. Treatment of BV-2 with minocycline before infection with *T.gondii* followed by co-culture with N2a for 24 h resulted in a dramatic reduction of TUNEL positive cells. Values represent the means ± SEM from three independent experiments. (*p < 0.01 *vs.* Control; #p < 0.01 *vs. T. gondii*).

To investigate the *in vivo* neuronal apoptosis in TE and the potential therapeutic effect of minocycline, fluorescence immunohistochemistry and Western blotting were performed to reveal the neuronal damage. The caspase-3 positive cells were observed in cortex of TE mice (Figure 2). The expression of NeuN (a specific neuron marker) of TE mice was significantly down-regulated when compared with the control (Figure 2). Accordingly, the *in vitro* studies also indicated that a low level of apoptosis of N2a cells was observed following co-culture or infection of N2a cells with parasites for 24 h (data not shown), suggesting that *T.gondii* inhibits rather than facilitates neuronal apoptosis. Apoptosis of N2a cells, however, dramatically increased when N2a cells were co-cultured with BV-2 cells and the tachyzoites for 24 h (Figure 3). As expected, minocycline treatment significantly attenuated cellular apoptosis of N2a in both *in vivo* and in *vitro* studies (Figures 2 and 3).

### Minocycline inhibited activation of microglia in cortex and hippocampus of mice with TE

**Figure 4:**
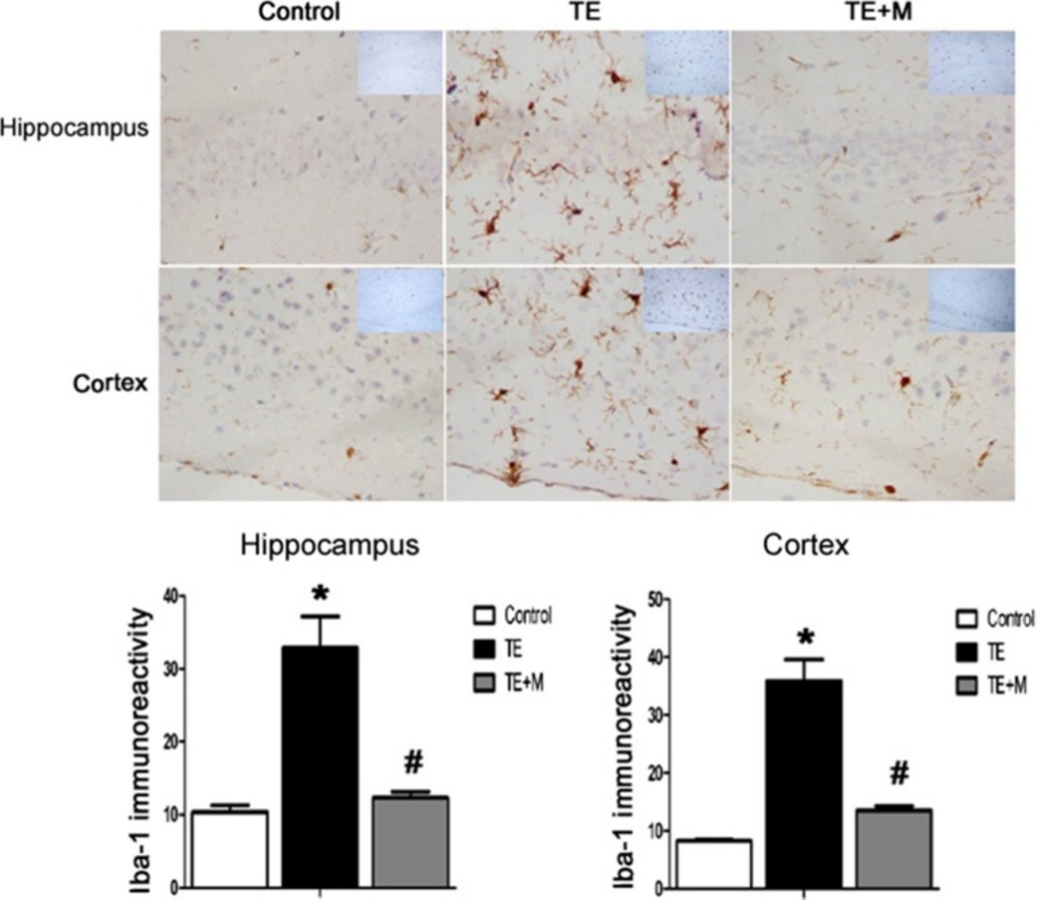
**Immunocytochemical analysis of microglia in the brain of TE and TE + M mice.** Brain sections from control, TE and TE + M animal groups were stained with polyclonal antibodies against Iba-1(a specific microglial marker). Compared with control and TE + M mice, the number of Iba-1-positive cells in both hippocampal and cortex of TE mice was remarkably increased. Morphological characteristics of microglia activation were noted in TE. Minocycline treatment significantly inhibited microglial activation. Values represent the means ± SEM of six animals per group. (*P < 0.01 vs Control; #P < 0.01 vs TE).

To investigate microglia activation in TE, antibodies against ionized calcium-binding adapter molecule-1 (Iba-1, a specific microglial marker) were used to label microglial cells. Immunohistochemistry analysis indicated that the number of Iba-1 positive cells was significantly higher in TE than in TE + M and control group (Figure 4). In the cortex and the hippocampus of TE mice, the microglia cells presented an extensively branched process and hypertrophy of cell body, representing the morphological features of cell activation (Figure 4).

### *T. gondii*induced secretion of inflammatory cytokines that were inhibited by minocycline

**Figure 5:**
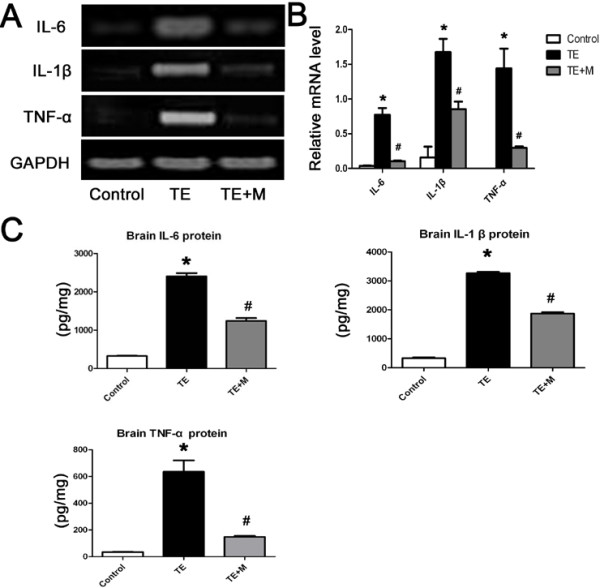
**Expression of pro-inflammatory cytokines (IL-1 β, IL-6 and TNF-α) in the brain tissues of control, TE, and TE + M mice. (A)** Representative agarose-gel photographs show the expression level of IL-1 β, IL-6, and TNF-α mRNAs in brain tissues tested by quantitative RT-PCR. **(B)** Histograms represent densitometric quantitation of IL-1 β, IL-6, and TNF-α, normalized over GAPDH mRNA expression. **(C)** Expression of IL-1 β, IL-6, and TNF-α in brain tissues examined by ELISA. Values represent the means ± SEM of six animals per group. (*, p < 0.01 *vs.* Control. #, p < 0.01 *vs.* TE).

Activated microglia contribute to the production of inflammatory cytokines that mediate direct or indirect neuron death. Herein, we analyzed the inflammatory cytokines generated by activated microglia cells challenged with *T.gondii* tachyzoites. The qRT-PCR analysis indicated that expressions of interleukin-1β (IL-1β), interleukin-6 (IL-6), and tumor necrosis factor-α (TNF-α) mRNAs was remarkably enhanced in mice with TE, especially of IL-1β and TNF-α (Figure 5A and B), and the corresponding result was noted in the translational level when compared with the uninfected control (Figure 5C).

**Figure 6:**
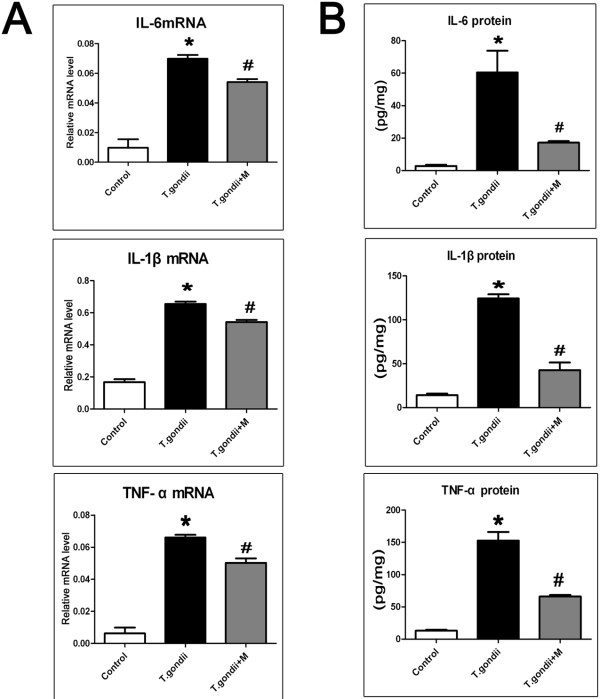
**Pro-inflammatory cytokines (IL-1 β, IL-6, and TNF-α) produced by BV-2 cells.** Mouse microglia BV-2 was either left uninfected, or *T.gondii*-infected (*T.gondii*) or *T.gondii*-infected and treated with minocycline (*T.gondii* + M) as described in the Methods section. **(A)** The mRNAs of pro-inflammatory cytokines were detected by quantitative RT-PCR. The relative levels of IL-1 β, IL-6, and TNF-α expression were compared with GAPDH. **(B)** Culture supernatants were collected after 24 h post-treatment and tested by ELISA to quantify IL-1 β, IL-6, and TNF-α secretion. The results represent the means ± SEM of three independent experiments. (*P < 0.01 vs Control; #P < 0.01 vs *T. gondii*).

To verify the *in vivo* data that the inflammatory cytokines were induced by activated microglia in TE, mouse microglial cells of BV-2 were subjected to infection with *Toxoplasma* tachyzoites, and IL-1β, IL-6, and TNF-α were detected with qRT-PCR and ELISA at 24 h post-infection. The results showed that expressions of IL-1β, IL-6, and TNF-α were significantly elevated following parasite inoculation (Figure 6). Minocycline treatment notably restrained release of IL-1β, IL-6, and TNF-α secreted by activated microglia in both *in vivo* and *in vitro* experiments (Figures 5 and 6)*.*

### *T. gondii*induced expression of iNOS in microglia which was blocked by minocycline

**Figure 7:**
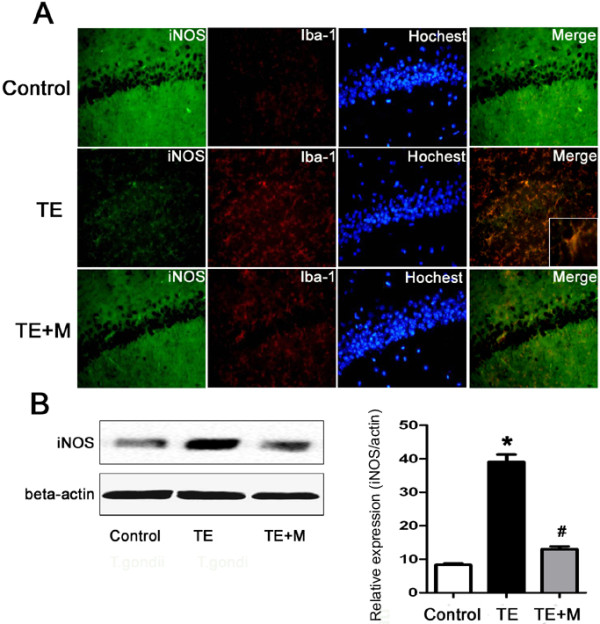
**Expression of iNOS in brain of control, TE, and TE + M mice. (A)** Double immunofluorescent staining of iNOS with Iba-1 (a specific marker of microglia). In the inflammatory loci of hippocampus, the number of iNOS-immunoreactive cells increased and the iNOS was expressed in microglia. **(B)** Western blotting analysis of iNOS expression. Histograms show densitometric analyses that revealed the relative levels of iNOS normalized over β-actin. Values represent the means ± SEM of six animals per group. (*P < 0.01 vs Control; #P < 0.01 vs TE).

Western blotting revealed a significant increase of expression of iNOS in TE mice compared with the uninfected group (Figure 7B). Double immunofluorescence staining demonstrated that iNOS was distinctly expressed by the Iba-1-immunoreactive cells rather than the neurons in the inflammatory foci (Figure 7A).

**Figure 8:**
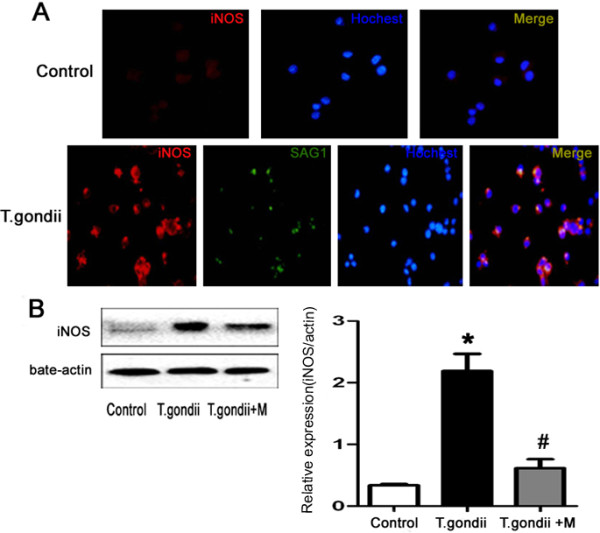
**Expression of iNOS protein in BV-2 cells.** The three groups include mouse microglia BV-2 cells (control), *T.gondii*-infected BV-2 cells (*T.gondii*) or BV-2 cells infected with *T.gondii* and treated with minocycline (*T.gondii* + M). **(A)** BV-2 cells were fixed and stained with antibodies against iNOS (red) and tachyzoite surface antigen SAG-1(green) 24 h after infection with *T.gondii*. Hoechst dye was used to stain nuclei (blue). Double immunofluorescence showed iNOS expression in parasite-infected BV-2 cells. **(B)** Western blotting analysis of iNOS. Histograms show densitometric analyses of the relative level of iNOS protein normalized over β-actin expression. Values represent the means ± SEM of six animals per group. (*P < 0.01 vs Control; #P < 0.01 vs TE).

Immunofluorescence and Western blotting were used to detect the *in vitro* expression of iNOS in BV-2 cells challenged with tachyzoites(Figure 8). Similar to the *in* vivo approach, tachyzoites facilitated activation of microglia to express iNOS, while minocycline treatment significantly reduced iNOS production following *T.gondii* infection*.*

## Discussion

Encephalitis caused by *T.gondii* is one of the lethal consequences in HIV/AIDS patients, and other immunocomprised individuals as well. So far knowledge has remained limited on the contributions of microglia to the pathogenesis of neuron damage in recrudescent toxoplasmosis. Results in the present study demonstrated that *T. gondii* induced microglia activation which up-regulated pro-inflammatory cytokine expression and mediated neuronal death. Treatment with minocycline not only inhibited microglia activation and the expression of pro-inflammatory cytokines, but also attenuated the neuronal apoptosis. Our data suggest that microglia activation might play a crucial role in the pathogenesis of Toxoplasmic encephalitis (TE).

We hypothesized in the present study that neuron apoptosis noted in TE might not be, at least in part, a result from the primary injury by parasitism but from the secondary response to microglia activation, which gives rise to the subsequent neuron cell damage, and is closely associated with the TE symptoms and signs [31].

Clinically, patients with TE exhibit severe neurological manifestations [32]. Previously, we found that reactivation of a latent *Toxoplasma* infection induced by cyclophosphamide led to hemiparesis and convulsions of mice, the pathological mechanism, however, remains unclarified. Although *T.gondii* readily invades neuron cells *in vitro* [33], interestingly, when N2a cells were co-cultured or infected with tachyzoites, only a small ratio of apoptotic cells were detectable. Previous investigations have shown that *T. gondii* could inhibit apoptosis of infected cells to ensure better dissemination within the host [4] and their own intracellular survival [34]. Several other studies, however, reported that *T. gondii*-infected cells induced apoptosis of bystander cells by secretion of some soluble factors [21, 35]. It is speculated that microglia may be involved in neuronal apoptosis in TE. As the resident macrophages of the CNS, microglia have extreme sensitivity to brain microenvironment stimuli, which responds to pathogen invasion or tissue damage by altering their morphology and phenotype. Similar to classically activated macrophages infected with Wh6 strain of type Chinese 1 [36], activated microglia can secrete a variety of pro-inflammatory and neurotoxic factors (e.g., IL-1β, IL-6, and TNF-α) and increase expression of iNOS. These factors are necessary to destroy invading pathogens and could directly induce apoptosis of host cells. Additionally, microglia activation is thought to be a causative factor in many neurological diseases including Alzheimer’s disease, Parkinson’s disease, and viral encephalitis [37]. These neural pathogeneses are generally associated with inflammatory mediators that are manifested in part by activated microglia. Relatively few studies have investigated the role of microglia in the pathogenesis of Toxoplasmic encephalitis. Herein, we observed that the activated microglia,as assessed by Iba-1 immunoreactivity,is increased dramatically in the cortex and hippocampus with TE, which positively correlates with the dominant distribution of *T. gondii* cysts in the cortex and hippocampus of the mouse brain [38, 39]. (Figures 1 and 4). Hippocampus and cortex are associated with cognitive and movement functions. Effects of *Toxoplasma* infection on human and animal behavior have been reported [40]. Here we noted the morphological alteration of microglia and increased expression of IL-1β, IL-6, TNF-α in *T.gondii* infected microglial cells and, by using transwell culture, we also found that the apoptosis deteriorated when N2a cells were co-cultured in the presence of microglia and parasites (Figure 3). These findings may provide some explanations for the neural symptoms and signs of hosts with Toxoplasmic encephalitis, although the role of *Toxoplasma* effectors (e.g., dense granule protein 15, GRA15) in polarization of microglia remains to be elucidated in the isolates of genotype Chinese 1.

Minocycline is a semisynthetic tetracycline that has been shown to have neuroprotective effects in animal models of various neural disorders [41, 42]. Minocycline is able to inhibit activation of macrophages and microglia, and thereby decrease inflammatory factor production (such as TNF-α, IL-1β, and IL-6) [43]. The present investigation indicates that minocycline significantly restrained microglia activation and, consequently, protected the neural cells from apoptosis *in vivo*. Additionally, minocycline remarkably reduced expression of IL-1β, IL-6, TNF-α, and iNOS. Apoptosis of N2a cells was significantly ameliorated when the co-cultured BV-2 cells were treated with minocycline. Furthermore, no statistically significant difference was seen between TE and TE + M groups in parasite burden in the brain of mice, suggesting the inability of minocycline to directly affect the vitality of *T.gondii*.

## Conclusions

Taken together, our findings reveal that *T.gondii* tachyzoites can strongly induce the secretion of inflammatory cytokines via activation of microglia both *in vivo* and *in vitro*. Inflammation generated by activated microglia is responsible for neuronal lesions in the brain of mice with reactivated Toxoplasmic encephalitis. The therapy of minocycline would help attenuate the pathology of mice with TE via inhibition of microglia activation and the causative cytokines involved.

## Authors’ information

Yi-hua Zhang and He Chen are co-first authors.

## Abbreviations

TE: Toxoplasmic encephalitis
CNS: Central nervous system
IL-6: Interleukin-6
IL-1β: Interleukin-1β
TNF-α: Tumor necrosis factor-α
iNOS: Inducible nitric oxide synthase
TBI: Traumatic brain injury
ICH: Intracerebral hemorrhage
LNB: Lyme neuroborreliosis
LPS: Lipopolysaccharide
GPIs: Glycosylphosphatidylinositols
ROPs: Rhoptries
GRAs: Dense granules.
